# Breeding matters: Natal experience influences population state-dependent host acceptance by an eruptive insect herbivore

**DOI:** 10.1371/journal.pone.0172448

**Published:** 2017-02-16

**Authors:** Jordan Lewis Burke, Allan L. Carroll

**Affiliations:** Department of Forest and Conservation Sciences, Faculty of Forestry, The University of British Columbia, Vancouver, British Columbia, Canada; University of Innsbruck, AUSTRIA

## Abstract

Eruptive forest insects are highly influential agents of change in forest ecosystems, and their effects have increased with recent climate change. State-dependent life histories contribute significantly to the population dynamics of eruptive forest insect herbivores; however, the proximate mechanisms by which these species shift between states is poorly understood. Laboratory bioassays were conducted using the mountain pine beetle (*Dendroctonus ponderosae*) to determine the effect of maternal host selection on offspring host preferences, as they apply to population state-dependent behaviors. Female mountain pine beetles exhibited state-dependent preference for artificial host material amended with monoterpenes in the absence of other cues, such that individuals reared in high-density epidemic-state simulations rejected low monoterpene conditions, while low-density endemic-state beetles accepted low monoterpene conditions. State-specific behavior in offspring was dependent on rearing conditions, as a function of maternal host selection, and these effects were observed within one generation. Density-dependent host selection behaviors exhibited by female mountain pine beetle offspring is reinforced by context-dependent maternal effects arising from parental host selection, and *in situ* exposure to conspecifics. These results demonstrate potential proximate mechanisms that control population dynamics in eruptive forest insects, and will allow for more accurate predictions of continued impact and spread of these species.

## Introduction

Herbivorous insects are the primary biotic disturbance agents within most coniferous forests. Whereas the majority of populations are highly regulated and are not prone to dramatic fluctuations in size [1], some intermittently experience widespread population eruptions and can inflict biome-scale disturbances [2,3]. For eruptive species, the constraints on individuals from sub-outbreak (i.e., endemic) populations are often very different than those associated with outbreak (i.e. epidemic) populations [3,4]. For example, strategies for dispersal, host selection, and mate location may differ by necessity when populations are low, and conspecifics rare, as compared to high-density populations where conspecifics are abundant. State-dependent life history strategies [5–10] have been demonstrated among diverse insect species with distinct population phases [11–14]. Although it is clear that the evolution of population-state dependent behaviors is an ultimate adaptation to extremes in population density [15], for most eruptive forest insect species the proximate mechanism(s) by which individuals shift between endemic and epidemic strategies/behaviors remains unclear.

Maternal effects, defined as causal influences of the maternal genotype or phenotype on offspring phenotype [16], have been implicated in population dynamics of many taxa (reviewed by [17]). For example, nutritional quality of food resources available to mothers may influence the health and fecundity of offspring [18], and maternal choices may change offspring growth rate or behaviors [19–21]. However, their precise role remains controversial since fluctuations in species abundance may have many causes [1,22]. Recent evidence suggests that the expression of maternal effects may be context dependent, influencing different traits and/or creating unique patterns of trait expression under different conditions [23]. For example, the influence of maternal size, age, or nutritional status on offspring fitness has been shown to depend on population density for a broad range of taxa [23–27]. Context-dependent maternal effects may be critical to the eruptive dynamics of species with population state-dependent life history characteristics by reinforcing traits specific to endemic or epidemic populations. For instance, if cooperative attacks of well defended hosts, characteristic of many epidemic-state bark beetle species [3], are associated with a density-dependent maternal effect, then the cooperative epidemic strategy will be reinforced across generations via enhanced access to additional well defended hosts associated with increased population density (i.e. positive feedback). Given evidence that the frequency and severity of outbreaks by some eruptive forest insects may be increasing due to climate change and other anthropogenic modifications to the environment [28,29], a clear understanding of the complex constraints on endemic and epidemic phases of these species is required to predict and mitigate impending outbreaks.

Mountain pine beetle (*Dendroctonus ponderosae* Hopkins) is an eruptive bark beetle (i.e. subcortical herbivore) that displays distinct endemic and epidemic population phases in pine forests of western North America [3,11,30]. The endemic state is the normative condition for the mountain pine beetle, and is characterized by very low-density populations (several females per hectare of forest) that preferentially colonize defensively impaired trees, that are often occupied by interspecific competitors [11,30,31]. When conditions facilitate an increase in generation survival, allowing numbers to increase beyond the threshold density for successful cooperative (i.e., mass) attack of large-diameter, healthy trees [30], beetles undergo a density-dependent shift in host preference and will selectively colonize highly defended trees [11]. Unique life-history strategies are associated with each population state. Endemic beetles must locate exceedingly rare and ephemeral, susceptible (i.e. defensively impaired) trees that comprise nutritionally suboptimal resources (thin phloem) [32], and then contend with potentially high levels of interspecific competition [30,33]. By contrast, virtually all mature pine trees are susceptible to epidemic beetles; however, the beetles must cope with the aggressive defensive chemicals produced by healthy trees, and accommodate high levels of intraspecific competition that results from mass attacks [30]. Cooperative mass attacks by epidemic beetles are facilitated by a complex synergism of host-produced (kairomones) and beetle-produced (pheromones) volatile chemicals [34], and by the introduction of mutualistic phytopathogenic ophiostomatoid fungi and various bacteria and other microbes during colonization [35–38]. Although the shift between endemic and epidemic behaviors by the mountain pine beetle is derived from changes in population density [11], the mechanism(s) by which beetles determine the appropriate behaviors and strategies have not been thoroughly investigated.

The threshold population density that defines the transition between endemic and epidemic states for the mountain pine beetle is determined by the defensive capacity of healthy host trees with thick phloem [30]. For *Pinus* spp., defenses against bark beetles rely primarily on the production of secondary metabolites, most notably monoterpenes [39–41]. Field studies suggest that foraging female mountain pine beetles assess monoterpene content gustatorially to determine host quality, quantify defensive capability, and accept or reject a potential host [11,42–44]. Given that the choice of a host by female insect herbivores comprises a potential maternal effect [16], and for the mountain pine beetle that choice varies based on population state [11], we investigated the role of maternal host choice (i.e. the state-dependent strategy) and offspring developmental experience (i.e. the context-dependent maternal effect) on subsequent host preference by mountain pine beetle. More specifically, using laboratory experiments we critically evaluated the hypothesis that host acceptance by foraging mountain pine beetles will be influenced by density-dependent maternal host choice. We predicted that the progeny of low-density females developing in poorly defended hosts (i.e. endemic behavior) will accept substrates indicative of low host defenses, whereas the progeny of high-density females from well defended hosts (i.e. epidemic behavior) will not.

## Materials and methods

### Mountain pine beetle collection

In September of 2012, 2013, and 2014, several naturally mass-attacked lodgepole pine trees were felled from epidemic infestations near Lillooet (N 50.684°, W 121.936°), Baldy Mountain (N 49.110°, W 119.117°), and Grand Forks (N 49.029°, W 118.445°), southern British Columbia, Canada. Permission to remove material was provided by the British Columbia Ministry of Forests, Lands, and Natural Resource Operations. These were cut into ca. 75-cm-long bolts and transported to the Forest Insect Disturbance Ecology Laboratory (FIDEL) at The University of British Columbia in Vancouver. The ends of the bolts were sealed with paraffin wax to prevent desiccation, and the bolts were placed in cages (2m × 2m × 1m wooden boxes, with 0.5 mm screen side panels) at room temperature until beetles completed their development and emerged (~4–6 weeks). Beetles were collected from the sides and top of the cages, segregated by sex based upon stridulation [45,46], and either assigned to an endemic or epidemic population state simulation as described below.

### Endemic simulation

Given that endemic mountain pine beetle populations are characterized by isolated attacks on vigor-impaired, small-diameter trees with thin phloem [30,31], these conditions were recreated in the laboratory to produce beetles with the potential for endemic behavior. Several small (<20 cm diameter at 1.3 m) lodgepole pine trees were harvested in the autumn from an uninfested stand near Whistler, British Columbia (N 50.115°, W 122.957°), cut into 30-cm-long bolts, sealed with paraffin wax and transported to the laboratory. In the lab, one 8-mm hole was punched through the bark near the bottom of each bolt. A newly emerged female beetle was placed into a 2 mL Eppendorf® vial (Fisher-Scientific®) with the end snipped off and the open end placed into the hole, such that the female could initiate tunneling beneath the bark. Vials were left in place for 24 hours and then assessed for the presence of boring dust indicative of successful entry. Females that did not enter were replaced by another female for a new 24-hour period, until successful. Once females had successfully established in the bolts, one male was rechecked for stridulation, then placed into the gallery entrance, where they immediately entered and initiated mating. Only one mating pair was introduced into these suboptimal (i.e. small-diameter) bolts, thereby simulating maternal host choice typical of endemic population phases.

Infested bolts were placed in individual endemic simulation chambers created from a 19 L (30 cm dia × 36 cm ht) bucket (The Home Depot®) with six, 5-cm-diameter mesh-covered holes on the sidewall. One bolt was placed in the bucket, and then the top was covered with screening and secured with string. Buckets were placed in individual rooms (≥10 m apart, separated by multiple walls) in the University of British Columbia Forest Sciences Centre to keep them isolated from each other and minimize the potential for interaction between non-sibling conspecifics during offspring development. Ambient temperature in all rooms was maintained between 20–22°C by the climate control of the building.

Endemic simulation chambers were checked three times daily to minimize contact among emerging beetles, and any beetles found were segregated by sex. Female beetles were immediately placed into individual 20 mL glass vials, containing a small piece of folded filter paper, to assist them in staying upright. In empty glass vials, beetles will often overturn and be unable to right themselves, and may die from exhaustion (J. Burke–personal observation). Males were either kept for use in other trials, or discarded, as host selection by mountain pine beetles is conducted exclusively by females [30]. Dispersing beetles will search for hosts for up to several days [47]. Therefore, to simulate foraging conditions for low-density populations, individual females in their glass vials were placed into 120 mL plastic specimen containers, along with about 10 lodgepole pine needles and 1 mL of distilled water, which were then sealed and placed in a fume hood under natural light for 48 hours. Needles were provided as a cue to indicate that the beetles were in the vicinity of host trees. This method simulated the foraging period post-emergence, but without the potential for interaction with conspecifics.

### Epidemic simulation

Epidemic simulation was achieved using beetles from the source population. The bolts collected from the naturally mass-attacked trees (> 25 cm diameter at 1.3 m) from active infestations represented *in situ* epidemic maternal host choice. To ensure the potential for epidemic-state maternal effects to manifest in progeny, bolts were grouped within emergence cages in a single room, and beetles were collected every 48 hours for use in trials, to maximize their potential for interaction with conspecifics. Beetles were always collected from the top or the sides of the cage and never from the log surface or the floor to ensure they were vigorous and minimize the chance of collecting a newly emerged beetle that had not had a chance to interact with conspecifics.

### Simulated phloem

Semi-artificial phloem diet comprising denatured lodgepole pine phloem, agar, water, and synthetic monoterpenes was used to simulate the sub-cortical environment occupied by the mountain pine beetle. This approach was modeled after previous, successful experiments using other bark beetle-conifer systems [12,13,48,49]. Additional lodgepole pine bolts from mature trees collected from the Whistler stand were stripped of their outer bark, which was discarded, then of the phloem tissue beneath, using a draw knife. Harvested phloem tissue was submerged in liquid nitrogen, crushed into small (ca. 1x1 cm) pieces using a large mortar and pestle, and then quickly placed into a coffee grinder (Black and Decker® model CBG100S) and pulverized. Ground phloem was run through a 0.5 mm sieve, and then put into an autoclave at 105°C for 20 mins to remove the existing volatile monoterpenes [50]. A mixture of agar (Fisher Scientific® BP1423-500) and distilled water at 60 g/L was created, and 300 mL of agar solution was added to 200 g of ground phloem and mixed evenly. The resulting mixture was ~70% moisture, by weight. Moisture content was checked for each new phloem batch by weighing, then drying samples in a drying oven at 70°C for 24 hours and reweighing.

**Fig 1:**
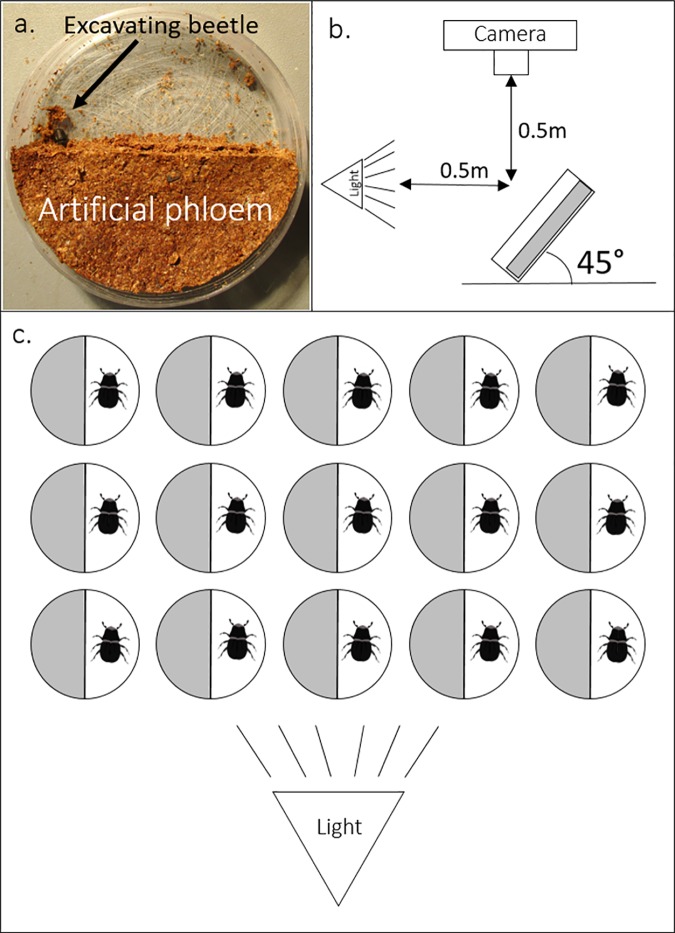
A photo of an arena and technical drawings of the setup of the assay used to determine the effect of population state on substrate acceptance by female mountain pine beetles; a) image of “half-moon” configuration of artificial diet in petri dish, with an excavating mountain pine beetle, b) configuration of arena, light, and camera for recording beetle activity, c) configuration of arenas and light during assay. Drawings are not set to scale.

Small (60 mm diam. × 15 mm ht.) Petri plates (Fisherbrand® FB0875713A) were textured on the inside using coarse-grit sandpaper to allow beetles to maneuver within the arena and achieve purchase on the plastic surface when boring into the simulated phloem. A triangular piece of wood (60 × 42 × 42 mm) was inserted into the arena, with the hypotenuse of the triangle bisecting the arena, and approximately 10 g of diet was pressed into the empty half. Prepared dishes were placed into a drying oven at 70°C for 1.5 hours, and after drying, the wood insert was removed (Fig 1A).

### Monoterpene blend

Mountain pine beetles can successfully reproduce in most North American *Pinus* species [51,52]. Consequently, beetles must contend with a range of monoterpenes during colonization. For example, two of the most common monoterpenes in defensive resin of pines, (+)- and (-)-α-pinene, comprise as much as ca. 50–90% of jack pine (*Pinus banksiana*) resin [53–59], but only ca. 7–12% of whitebark pine (*Pinus albicaulis*) resin [60]. A blend of monoterpenes was created to represent the major components present in potential *Pinus* hosts. A monoterpene solution containing 50% (-)-β-phellandrene distilled from lodgepole pine turpentine was obtained from C. Breuil at UBC. At the time of this experiment, β-phellandrene was not available for purchase in purified form. This chemical was required as it is the major monoterpene component of lodgepole pine phloem resin [53,58,61]. The solution contained additional monoterpenes that were intended to be used, so rather than distilling it further to isolate β-phellandrene, it was augmented with (+)- and (-)-α-pinene, (-)-β-pinene, myrcene, 3-carene, and limonene from Sigma-Aldrich® (www.sigma-aldrich.com). The final solution comprised the following component ratios: 32.6% racemic α-pinenes, 32.6% (-)-β-pinene, 3.8% 3-carene, 10.6% myrcene, 3.2% (-)-limonene, 15.3% (-)-β-phellandrene, and 1.9% terpinolene. (-)-β-pinene and (-)-β-phellandrene are major components of lodgepole pine resin [53], and the remaining components have various biological functions in this system; 3-carene and limonene are toxic to beetles [62], and myrcene and terpinolene are pheromone synergists [63]. Alpha-pinene is particularly important, as it is both detrimental [35] and beneficial [53,55,57,64] to foraging beetles. Aliquots of the monoterpene mixture were then diluted in pentane [50] to 10% and 50% (for 10 and 50 mg/g treatments), with the remaining left at 100% monoterpene concentration (for 100 mg/g treatments). Approximately ~115 μL of the appropriate monoterpene mixture (10, 50, 100%) was added per gram of simulated phloem (~700 μL) to the surface of the substrate in the petri dishes, allowing the liquid to penetrate and distribute evenly. Arenas were then left uncovered under a fume hood for 1 hour to allow the pentane to evaporate [50]. In this way, the same amount of liquid was added to each arena, while producing different concentrations of monoterpenes.

### Substrate acceptance *versus* population state

Once the arenas were prepared, one female mountain pine beetle offspring, either from the mass-attacked bolts maintained in the large emergence cages (epidemic simulation), or the smaller bolts in the isolated endemic simulation chambers, was placed into the empty side of an arena (Fig 1A). The lid of the petri dish was labeled with a marker and placed onto the arena. These were then placed onto a foam board, and secured standing at a 45° angle to the board with insect pins (Fig 1B). This configuration ensured that beetles could right themselves if they tipped over (J. Burke–personal observation). Between 6 and 8 replicates of each concentration treatment were used in each trial. A small fan was placed near the arena board to gently move air over the surface and ensure volatiles would not accumulate over the arenas. Each arena was angled in the same direction, facing a 120-watt equivalent, full spectrum LED flood lamp (Philips® model PAR38), to approximate daylight (Fig 1C). Due to the process of rearing, epidemic simulation beetles were tested first, then endemic simulation beetles were tested ca. 4–6 weeks later, under identical conditions.

Once all beetles and arenas were in place, the arena board was situated underneath a Logitech® HD Pro C920 webcam video camera, which recorded beetle activity for 8 hours (Fig 1B). To clearly see the beetles, the camera was set to capture in greyscale (0% color saturation) and low color contrast. The black beetles were clearly visible against the grey and white background of the arena and foam board under these recording conditions. A time-lapse series of photographs was created from still images using Chronolapse® software version 1.0.7 (2010, Collin Green, https://code.google.com/p/chronolapse/), which is an open-source software application (http://opensource.org/licenses/mit-license.php). A picture was recorded every 30 minutes for 8 hours, at 2304 x 1296 resolution. A beetle was considered to have accepted the simulated phloem when she had entered it completely and her entire body was no longer visible. Preliminary, 24-hour trials revealed very little change in acceptance/rejection after 8 hours, so all trials were stopped at this time-point. At the end of each trial, the arenas were frozen at -30°C for 48 hours, and the dead beetle was excavated and the width of her pronotum was measured. The elytra were then removed and each beetle was checked for sexually dimorphic characteristics (*sensu* [65]) to confirm the subject was female. Of 292 total beetles trialed over the course of the experiment, only 4 were males (that did not stridulate), and these were removed from the analysis.

In total, 38 trials were run with three concentration treatments. Seven trials of 8 beetles per concentration treatment were run with beetles from epidemic simulations (168 beetles). Number of trials run per concentration treatment and beetles per trial varied within endemic simulation, as rearing beetles at such low density limits the number of offspring available to trial at any one time. Six trials of 6–8 beetles were run for 10 and 50 mg/g treatments, and 5 trials of 8 beetles were run for 100 mg/g treatments (124 beetles).

### Statistical analyses

All data were prepared using Microsoft® Excel® version 16.0 (Microsoft®, 2015, Redmond WA USA), and all statistical tests were performed using SAS® Statistical Software version 9.4 (SAS® Institute, 2014, Cary NC USA). The mean acceptance ratio (entered/non-entered) was calculated for each trial of 6–8 beetles, at each hour of the eight hour trials (n = 38 × 8). All data were arcsine(square-root) transformed to account for the truncated distribution of proportion data [66]. PROC UNIVARIATE was applied to test the assumptions of normality and equality of variance.

Two-way analysis of variance with interactions was performed using PROC GLM, with population-state simulation and concentration treatments as main effects, on the acceptance ratio after 8 hours. To more thoroughly examine the influence of juvenile rearing conditions on subsequent adult host acceptance, PROC GLM was used to assess variation in substrate acceptance by simulated endemic and epidemic beetles within each concentration treatment after 8 hours.

To assess the effect of developmental experience on the rate of acceptance, a repeated-measures analysis of variance was performed on data in time-series from 1–8 hours of exposure, for each concentration treatment using PROC GLM with the REPEATED statement. The interaction of time (i.e. “rate”) and simulation was assessed to determine if the rate of acceptance differed between simulations, within each concentration treatment.

PROC GLM was used to perform one-way analysis of variance to determine if simulation treatment affected offspring size. Since the thin phloem of defensively impaired trees may lead to smaller offspring [32,65], and small beetles may be less able to overcome defenses of larger, healthy hosts [67], the effect of body size (pronotal width) on beetle acceptance was assessed using logistic regression (PROC LOGISTIC) on the original, binary (0/1) data at 8 hours, within simulation, and within concentration treatments, to determine if larger beetles were more likely to accept high monoterpene substrates.

## Results

**Fig 2:**
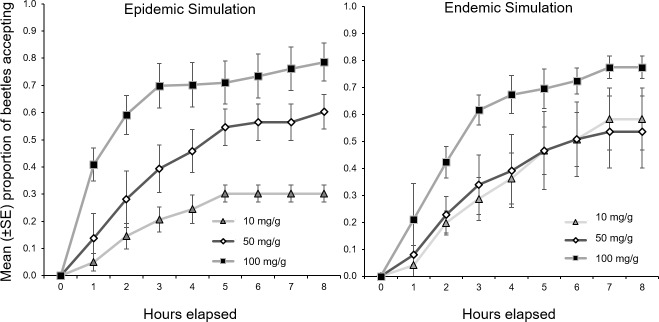
Mean (±SE) acceptance ratio (accept/reject) per trial (38 trials, 292 total beetles) of simulated phloem amended with three monoterpene concentrations by female mountain pine beetles reared in two population-density simulations over the course of 8-hour trials.

When considered all together, female mountain pine beetles accepted simulated phloem about 50% of the time (Fig 2). Approximately 85% of beetles that accepted the simulated phloem did so after 5 hours. Those that did not initiate tunneling spent most of their time walking on the surface of the simulated phloem and in the empty side of the arena. Three beetles remained motionless, and upon inspection after 8 hours were found to have died during the trial and were removed from analyses. Once initiated, tunneling activity lasted for about 2–3 hours (observed by accumulating material at the entrance site). Occasionally, beetles emerged from the simulated phloem, likely due to limited tunneling space, but these were still considered to have accepted the diet.

**Fig 3:**
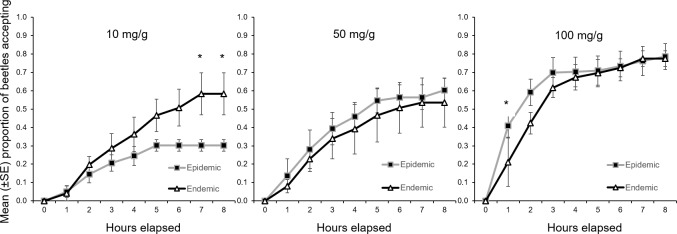
Mean (±SE) acceptance ratio (accept/reject) per trial (38 trials, 292 total beetles) of simulated phloem amended with three monoterpene concentration treatments by female mountain pine beetles, reared in two population-density simulations over the course of 8-hour trials. Asterisks (*) indicate significant differences (p<0.05) determined by analysis of variance.

The concentration of monoterpenes within the simulated phloem significantly affected the likelihood that a female beetle would mine within it (F_2,37_ = 6.69, p = 0.004) (Fig 2, Table 1). Overall, beetles were more likely to accept the phloem with the highest concentration (100 mg/g) over lower concentrations at all time-steps, and within the epidemic simulation acceptance ratio was directly related to monoterpene concentration at all time-steps (Fig 2). Although there was no overall effect of beetle rearing condition on acceptance of the phloem substrates among monoterpene concentrations, nor a detectable interaction between simulated population state and concentration (Fig 3, Table 1), acceptance of the phloem substrate with the lowest monoterpene concentration (10 mg/g) differed significantly between epidemic and endemic beetles (F_1,12_ = 6.24, p = 0.03). The effect of population state did not persist at the higher monoterpene concentration levels (Fig 3, Table 1).

**Table 1:** Summary statistics for analysis of variance tests of the effect of monoterpene concentration and simulated population state on total acceptance ratio of simulated phloem by female mountain pine beetles, after 8 hours, and for tests segregated by concentration treatment. Significant (p<0.05) effects are shown in ***bold italics*.**

*Source*	*df*	*Type III SS*	*F*	*p*
Monoterpene concentration	2	0.983	6.05	***0*.*006***
Simulated population state	1	0.023	0.29	0.592
Concentration*simulation	2	0.299	1.84	0.175
Total	37			
*10 mg/g*				
Simulated population state	1	0.293	6.24	***0*.*030***
Total	12			
*50 mg/g*				
Simulated population state	1	0.016	0.13	0.723
Total	12			
*100 mg/g*				
Simulated population state	1	0.012	0.17	0.686
Total	10			

The effect of time (rate) was always significant within concentration treatments (p<0.001) (Table 2). Furthermore, at the lowest and highest monoterpene concentrations, the rate of acceptance was dependent upon simulated population state. The rate of acceptance by endemic beetles of 10 mg/g treatments was significantly higher (F_7,77_ = 2.15, p = 0.498), while their rate of acceptance of 100 mg/g treatments was significantly lower (F_7,63_ = 3.77, p = 0.002) than that of epidemic beetles (Fig 3, Table 2).

**Table 2:** Summary statistics for repeated measures analysis of variance tests on the effect of simulated population state on the rate of acceptance of simulated phloem amended with different monoterpene concentrations by female mountain pine beetles after 8 hours. Significant (p<0.05) effects are shown in ***bold italics*.**

*Source*	*df*	*Type III SS*	*F*	*p*
*10 mg/g*				
Rate	7	4.24	29.19	***<0*.*001***
Simulation	1	0.63	2.45	0.146
Rate*simulation	7	0.33	2.15	***0*.*049***
Error(rate)	77	1.55		
*50 mg/g*				
Rate	7	4.37	19.51	***<0*.*001***
Simulation	1	0.11	0.14	0.711
Rate*simulation	7	0.01	0.04	0.999
Error(rate)	77	2.47		
*100 mg/g*				
Rate	7	3.87	35.55	***<0*.*001***
Simulation	1	0.58	1.19	0.304
Rate*simulation	7	0.41	3.77	***0*.*002***
Error(rate)	63	0.98		

Pronotal widths ranged from 1.8–2.6 mm with a mean of 2.27 (± 0.01 SE). Contrary to expectation, body size of beetles from each simulation were not different (p = 0.65), and there was no effect of beetle body size on the likelihood of acceptance of phloem at any monoterpene concentration (p = 0.64–0.96).

## Discussion

State-dependent life history strategies are influenced by context-dependent maternal effects in the mountain pine beetle. The propensity for maternal experience to influence the pattern of offspring host acceptance within population states can be used to elucidate critical aspects of herbivore-tree interactions that underlay eruptive population dynamics. Initial host selection by female mountain pine beetles begins with foraging individuals randomly landing on host trees [68], followed by gustatory assessment of resin components upon initial breach of the outer bark [44]. A beetle will then accept or reject that host as a function of population state, and putatively, the concentration of monoterpenes in phloem resin [11]. In bioassays, decisions regarding host acceptance were made based on monoterpene concentrations in phloem tissue even in the absence of other cues. This supports the assertions of Raffa and Berryman [44] that beetles use gustatory stimulation to assess host tree condition, and Boone et al. [11] that density-dependent host selection by the mountain pine beetle is based on assessment of monoterpene concentration in resin, and that this association is not an artifact of other search criteria employed by foraging beetles. The result that population-state simulation changed acceptance patterns further reinforces the assertion by Boone et al. [11] that their preferences for low or high concentrations are a function of population-state.

Contrary to expectations, beetle body size had no influence on host acceptance. Previous work has shown that mountain pine beetle body size is positively related to phloem thickness of natal host trees [32,65,69], and has potentially important ramifications to beetle fitness through its influence on energy reserves [70], and ability to detoxify host-tree defenses [67]. Therefore, the absence of a positive relationship between body size and acceptance of increasingly defensive substrates was surprising. However, parental beetles used in this investigation were on average larger and not representative of the full range of sizes observed in previous assessments that considered mountain pine beetle body size [67,71], and therefore any effect of body size may have been masked. The potential for reduced offspring body size resulting from development in nutritionally suboptimal hosts would likely reinforce their preference for low concentrations of monoterpenes, given the relationship between size and detoxification potential [67]. A similar mechanism was proposed by Ginzburg and Tanneyhill [18] as a regulator of population cycles in forest Lepidoptera.

Variation in the likelihood of acceptance of host-tree defenses as a function of maternal experience provides valuable insights into the state-dependent life histories of the mountain pine beetle and other eruptive bark beetles, and the transition between states leading to population eruptions and collapse. Maternal endemic behavior (isolated attacks of suboptimal hosts), results in offspring that are more likely to accept poorly defended substrates, whereas maternal epidemic behavior (mass attacks of healthy trees) leads to offspring that are more likely to reject poorly defended substrates. This suggests that in nature, offspring from mothers which select impaired hosts will have a greater propensity to also select an impaired host, increasing the chances of persistence over local extirpation in low-densities due to the strong Allee effects associated with a threshold density required to attack healthy trees [72–74]. Endemic offspring were slow to accept, but did not reject defensive substrates after 8 hours. However, this may be due in part to the feeding-stimulant properties of monoterpenes [44] combined with the fact that beetles could not leave the arena. Regardless, this variation may have implications for the transition from the endemic to the epidemic phase. During the endemic phase, if generation mortality is reduced due to a density-independent perturbation such as mild winter temperatures or drought leading to reduced tree defensive capacity [75,76], then offspring will interact with more conspecifics during development, potentially triggering epidemic behavior and attacks on defensive hosts. Furthermore, phenotypic plasticity in the response of endemic offspring may lead to a synergistic reinforcement of this transition by those offspring with the capacity to accept well-defended hosts, thus facilitating the rapid transition from endemic to epidemic population-phase characteristic of mountain pine beetle dynamics [11,30].

In contrast, most offspring from epidemic mothers rejected low concentrations of monoterpenes, and those that did accept were slow to do so, even when given no choice. This means that an epidemic population *in situ* has a very strong propensity to persist, and is resilient to collapse even following an extreme density-independent mortality event such as an extremely cold winter [30]. The strong reinforcement of epidemic host selection by maternal experience is the proximate mechanism behind positive feedbacks associated with this population phase at the stand and landscape scale [3]. Offspring of epidemic parents are most likely to pursue a well-defended, nutritionally optimal host under most conditions. If optimal hosts are abundant, reinforcement of the epidemic choice will result in many of these hosts being colonized, increasing the potential to colonize even more optimal hosts in the following year, rapidly leading to a stand-level outbreak. If optimal stands are abundant on the landscape, beetles dispersing into adjacent stands will take with them a strong propensity for epidemic selection, even if they initially arrive without many conspecifics. If enough beetles join them, aggregation will be successful, the stand-level epidemic process will initiate, and the outbreak will propagate. Thus, reinforcement of epidemic-phase host selection behavior by context-dependent maternal experience can lead to a landscape-scale disturbance event.

Strong reinforcement of epidemic behavior by maternal experience also means mountain pine beetle populations will rapidly deplete the local supply of optimal hosts and collapse; a common observation during epidemics [30,37,65,77,78]. Eventual host depletion means it is inevitable that a generation of mothers will find themselves without large trees to attack or insufficient conspecifics to aggregate, and be at high risk for local extinction. Given that maternal experience influences offspring host acceptance, the propensity for endemic behavior will increase and populations will transition back to the endemic state. Indeed, this transition appears to happen very quickly. Endemic mothers employed in experiments were reared as epidemic offspring until introduced into defensively impaired logs in isolated conditions, leading to endemic behavior in their offspring. Thus, the influence of population state-dependent maternal effects can manifest in a single generation to both minimize the likelihood of local extirpation, and maximize the potential for outbreak.

Evidence for the influence of context-dependent maternal effects on mountain pine beetle eruptive dynamics can be extended to the consideration of the impacts of climate change on landscape-scale forest disturbances by other eruptive forest insect systems. Herbivorous forest insects in Holarctic regions often do not occupy the more northern range of their hosts, meaning that the potential for native invasion is high [78–81]. Indeed, increasing temperatures have led to range expansion and shifts into novel habitats for several eruptive forest insects [79,82–84]. Furthermore, novel habitats may exhibit insufficiently coevolved defensive traits that increase host susceptibility [53,55–57,60,77]. Weak host defenses may lead to reduced generation mortality of eruptive insects, increasing the propensity for epidemic behavior, enhance positive feedbacks associated with this phase, and exacerbate the frequency and magnitude of outbreaks. A more thorough understanding of the proximate causes leading to eruptions is required to improve predictions and mitigate the biological consequences of continued climate change.
